# How people with persistent pain experience in-person physiotherapy blended with biopsychosocial digital health - A qualitative study on participants' experiences with Back2Action

**DOI:** 10.1016/j.invent.2024.100731

**Published:** 2024-02-28

**Authors:** E.Y. Poolman, L. Vorstermans, M.H. Donker, L. Bijker, M.W. Coppieters, P. Cuijpers, G.G.M. Scholten-Peeters, L.M. de Wit

**Affiliations:** a Department of Human Movement Sciences, Faculty of Behavioural and Movement Sciences, Vrije Universiteit Amsterdam, Amsterdam Movement Sciences - Program Musculoskeletal Health, the Netherlands; b Amsterdam Public Health Research Institute, Department of Clinical Psychology, Faculty of Behavioural and Movement Sciences, Vrije Universiteit Amsterdam, the Netherlands; c Master Musculoskeletal Rehabilitation, HAN University of Applied Sciences, Nijmegen, the Netherlands; d Department of Health Sciences, Faculty of Beta Sciences, Vrije Universiteit Amsterdam, the Netherlands; e School of Health Sciences and Social Work, and Menzies Health Institute Queensland, Griffith University, Brisbane & Gold Coast, Australia

**Keywords:** Primary health care, Low back pain, Neck pain, Pain education, Behavioural activation, eHealth

## Abstract

**Background:**

A blended intervention consisting of in-person physiotherapy and psychologically-informed digital health, called Back2Action, was developed to optimise the management of people with persistent spinal pain who also have psychosocial risk factors associated with the development or maintenance of persistent pain. This study aimed to gain insights in how participants experienced this blended intervention.

**Methods:**

A qualitative study using semi-structured interviews was conducted. Eleven people with persistent non-specific spinal pain who received the blended intervention within a randomised clinical trial were included. All interviews were recorded, transcribed verbatim and analysed independently by two researchers. Data were analysed using a thematic analysis.

**Results:**

The analysis identified four themes: (1) Experiencing a better understanding of the relationship between own physical and mental health; (2) Importance of the physiotherapist's active involvement in biopsychosocial blended care, which describes the crucial role of physiotherapists in supporting participants in this; (3) Appreciation of digital health, to better understand persistent pain and make meaningful lifestyle changes; and (4) Trials and triumphs, revealing gains such as better coping, but also challenges with implementation of changes into long-term routines.

**Conclusion:**

Participants of the blended intervention experienced positive changes in thoughts and behaviours, which highlights the feasibility and acceptability of the blended intervention as a more holistic treatment within pain management. The differences in personal preferences for receiving psychologically-informed digital health poses challenges for implementation of blended biopsychosocial care in evidence-based practice.

## Highlights

• A biopsychosocial blended intervention allowed individuals to achieve success, such as re-engaging in meaningful activities.
• The integration of pain education and behavioural activation techniques strengthened the intervention's holistic approach.
• Physiotherapists’ active involvement played an important role in encouraging participants to complete the digital health modules.

## 1 Introduction

Non-specific low back pain and neck pain are often influenced by a complex interaction of psychological, social and physical aspects, as well as comorbidities and mechanisms related to pain processing (Hartvigsen et al., 2018; Kazeminasab et al., 2022). In particular, psychosocial factors, such as depression, anxiety and kinesiophobia, appear to be associated with impaired recovery from spinal disorders (Ramond et al., 2011; van't Land et al., 2011; Pinheiro et al., 2016).

People with persistent, non-specific spinal pain who also have psychosocial risk factors associated with the development or maintenance of persistent pain, are often treated by physiotherapists (Synnott et al., 2015). The use of a biopsychosocial model, an approach within healthcare that acknowledges health and illness result from complex interactions between biological, psychological, and social factors (Engel, 1977), is advocated in the physiotherapy pain management of these people (Hartvigsen et al., 2022; Ho et al., 2022; Miki et al., 2023). However, physiotherapists indicated that they do not always have the necessary skills and confidence to successfully address and treat the psychological problems seen in spinal pain (Synnott et al., 2015; Holopainen et al., 2020). Providing support in treating these psychological problems could empower them to truly deliver biopsychosocial care.

Recommended interventions for psychologically-informed care are pain education and behavioural activation (Mazzucchelli and Da Silva, 2016; Tegner et al., 2018; Vitoula et al., 2018). Pain education refers to educational interventions that aim to change a person's understanding and thoughts about their pain (Moseley and Butler, 2015). Behavioural activation is a psychological treatment which stimulates people to plan meaningful activities to enhance their mood (Walsh et al., 2022). Although there are indications that these interventions (i.e., pain education and behavioural activation) can be effective on multiple outcomes, such as pain, disability, anxiety and depression (Guerrero et al., 2018; Hall et al., 2018; Wood and Hendrick, 2019; Williams et al., 2020; Bülow et al., 2021), physiotherapists often do not have the necessary skills and confidence to address and treat the psychosocial risk factors seen in people with spinal pain (Synnott et al., 2015; Wijma et al., 2016; Holopainen et al., 2020).

To optimise the management of people with persistent spinal pain and to complement the skillset regarding psychologically-informed care for physiotherapists in primary care, a biopsychosocial blended intervention was developed called “Back2Action” (Bijker et al., 2022). This intervention consists of regular in-person physiotherapy sessions blended with psychologically-informed digital health, based on pain education and behavioural activation.

To successfully implement a newly developed intervention, adherence to evidence-based practice (EBP) is paramount. This means that not only should the intervention be grounded in the best available scientific evidence (Sackett et al., 1996), but it is also crucial to conduct research into the needs and expectations of participants (Neve et al., 2010; Kayser et al., 2015). By exploring participants' experiences, insights are gained into the useful elements of the intervention and areas that may require attention. Therefore, the research question of this qualitative study was: How do participants with persistent spinal pain experience this biopsychosocial blended intervention?

## 2 Methods

This study was a qualitative study regarding the experiences of participants who received the biopsychosocial blended intervention “Back2Action” (Bijker et al., 2022).

The checklist for reporting qualitative research (COREQ) was used to design and report the study, see appendices (Tong et al., 2007). The study was conducted alongside a randomised clinical trial (RCT) evaluating the efficacy and cost-effectiveness of Back2Action compared to usual physiotherapy care (Bijker et al., 2022) (International Clinical Trials Registry Platform (NTR6122)). The trial was conducted according to the principles of the Declaration of Helsinki (2013) and was approved by the Medical Ethical Committee (METC) of the VU Medical Centre Amsterdam, The Netherlands (2017.286). This qualitative study was approved by the Institutional Review Board of the Faculty of Behavioural and Movement Sciences (VCWE 2021–129) of the Vrije Universiteit Amsterdam. All participants provided written informed consent prior to participation.

### 2.1 Participants

The study sample consisted of people with at least six weeks of non-specific low back pain and/or neck pain who also experience psychosocial risk factors associated with the development or maintenance of persistent pain, such as depressive symptoms, anxiety and/or kinesiophobia. Recruitment took place among all people allocated to the experimental arm (*n* = 20) during the last year of the RCT (2021). These people received written information via email about the purpose of this study, the procedures for participating in scientific qualitative research were explained and they were informed about the interviewers' occupation and their interest in this study.

### 2.2 Back2Action – a biopsychosocial blended intervention

Back2Action is a newly developed biopsychosocial blended intervention consisting of.

in-person physiotherapy sessions blended with psychologically-informed digital health, aimed at addressing persistent spinal pain and coexisting psychosocial factors. The digital part of the intervention incorporates pain education (Moseley and Butler, 2017) and behavioural activation (Martell et al., 2013), and consists of six digital modules. Each module takes approximately 45 min to complete and includes written information, patient examples, videos, tasks to identify personal factors, quizzes with true/false questions, activity scheduling by planning value-based activities in a weekly schedule, and a pain and mood diary (Bijker et al., 2022).

In-person physiotherapy was provided by primary care physiotherapists in The Netherlands. Physiotherapists were instructed to follow the Dutch Clinical Practice Guidelines for low back pain (van der Wees et al., 2021) and neck pain (Bier et al., 2016) which are in line with international guidelines (Corp et al., 2021). First line recommended interventions from these guidelines include information, (pain)education, exercise therapy, behaviour-oriented treatments, and second line recommended interventions include treatments such as mobilization, or manipulation of the lumbar spine. They received a three-hour training and a protocol on how to integrate the content of the digital health modules with their physiotherapy sessions. Physiotherapists were instructed to provide written feedback on the completed digital health module before unlocking the subsequent module.

### 2.3 Data collection

Data were collected through semi-structured in-depth interviews. Interviews were conducted online through Zoom.us., an online meeting platform. Both interviewers, one female (EP) and one male (LV), were physiotherapists experienced in treating people with spinal pain and with experience in qualitative research. There was no pre-established familiarity between researchers and participants. Questions during the interviews were based on a pre-defined topic list derived from the literature (Beck, 1964; Dweck, 2006), content of the digital health modules and intuition of the researchers. The interviews began with questions about past experiences, like why participants sought physiotherapy and what their motivation was to join the Back2Action RCT. This transitioned into deeper discussions about expectations, reasons for continuing or discontinuing the digital health, perceived changes post-treatment, and factors influencing these changes. Finally, interviews covered more specific Back2Action topics, including experiences with the physiotherapist, digital health, and blended care. If each topic was discussed and the conversation did not yield new information, the interviews ended. After three interviews the process was evaluated and adjustments to the topic list were made. For instance, there was a shift from focusing less on past experiences, such as reasons for seeking physiotherapy, to placing more emphasis on future perspectives – what would you require in the treatment of your pain.

### 2.4 Procedure

Before the start of the interview, demographics (sex, age, educational level), number of completed modules, number of physiotherapy sessions, pain location and pain intensity at baseline were extracted from the RCT data. On the day of the interview, we collected information about duration of neck and/or back pain and the pain intensity on that day.

All interviews were recorded (audio and video) using Zoom, and by mobile phone as back-up. The audio recordings were transcribed verbatim using Amberscript and were checked manually by one researcher (EP or LV). A brief summary (member check) of the interview was sent to the participant within one week after the interview to give the participant the opportunity to correct misinterpretations and/or provide additional information.

### 2.5 Data analysis

Data were analysed inductively by two researchers (EP and LV) using a thematic analysis (Braun and Clarke, 2006). The first six interviews were read, re-read and coded independently (EP and LV) and were discussed during multiple peer debriefings with a qualitative research expert (MD). At this first stage, meaningful parts of the transcript were independently labelled (open codes), which were compared until consensus between the three researchers (EP, LV and MD) was reached. These open codes were printed, cut into pieces and stacks of similar codes were created. These stacks were put in small envelopes which were labelled. Subsequently, the small envelopes were bundled together and placed into lager envelopes, while searching for overarching connections and initial theme names were given. Based on these codes, small and large envelopes, a thematic map was constructed. After six interviews an ‘all-encompassing’ thematic map was developed and discussed with the entire research group until consensus was reached.

All codes derived from the data and were formed and analysed in a systematic, repetitive manner. The qualitative data analysis software MAXQDA (version plus 2020) was primarily used for organizing and documenting our data, rather than for data analysis. The following five interviews were added in MAXQDA by one researcher (EP or LV) and were checked by the other. No new themes emerged after these six initial interviews, but the existing themes became richer.

Multiple peer debriefings with the entire research group - qualitative data expert (MD), physiotherapists and persistent pain experts (GS, MC) and psychologists and depression/anxiety experts (LW, PC) - took place during the data collection and iterative data analysis process to ensure the quality of the analysis. An audit trail was kept throughout the whole study.

## 3 Results

Of the 20 eligible people, 9 declined due to lack of time or interest. Their characteristics did not differ from those included in the study. The median (IQR) age of the 11 included participants was 28 years (25–44) and 55 % was female. They completed a median (IQR) number of 4 (1–6) digital health modules and eight physiotherapists were involved in their management from seven different practices throughout The Netherlands. To enable to transferability of the data and interpret the quotes detailed information of the participant characteristics are presented in Table A.

**Table A:** Demographics and characteristics of the participants.

ID	Sex	Age in years	Educational level	Location of pain	Duration of pain	Number of modules completed	Number of physiotherapy treatments	NPRS at baseline	NPRS at day of interview
P01	Female	31–35	Higher vocational education	Neck and Back	1.5 years	6	6	8	2
P02	Female	26–30	University degree	Neck and Back	1.5 years	6	4	7	1
P03	Female	20–25	Secondary vocational education	Neck	2 years	6	7	8	7
P04	Male	20–25	Secondary vocational education	Neck and Back	15 years	4	6	8	1
P05	Male	26–30	Secondary vocational education	Neck	15 years	0	4	3	0
P06	Male	31–35	Secondary vocational education	Neck and Back	1 year	1	9	1	3
P07	Male	20–25	Secondary vocational education	Neck and Back	5 months	1	13	7	7
P08	Female	20–25	Secondary vocational education	Neck and Back	6 months	1	5	5	3
P09	Male	41–45	Higher vocational education	Back	2.5 years	6	8	3	Missing
P10	Female	61–65	Higher vocational education	Neck and Back	20 years	6	6	7	2
P11	Female	46–50	Higher vocational education	Neck and Back	15 years	3	3	8	7

The interview duration varied between 30 and 60 min, depending on the richness of conversation and resulted in four main themes: (1) Experiencing a better understanding of the relationship between own physical and mental health; (2) Importance of the physiotherapist's active involvement in biopsychosocial blended care; (3) Appreciation of digital health; and (4) Trials and triumphs.

### 3.1 Theme 1: experiencing a better understanding of the relationship between own physical and mental health

Participants' attitudes towards psychologically-informed care influenced their willingness to embrace or reject Back2Action. Acknowledging that an altered mood, stress, or anxiety could potentially prolong their pain proved challenging for participants initially, as it contradicted their belief that their pain was solely physical in nature.

P03: “Initially, I wasn't immediately convinced when the physiotherapist suggested that my back pain was not solely caused by issues in my back. It's not that I instantly dismissed it, but then I started to consider it more carefully and realized that my own stress and worries could also be contributing factors. Once the physiotherapist brought this to my attention it was a revelation, and made me realize my back pain could potentially improve in the future.”

While Back2Action helped many participants to understand the relationship between their physical and mental health, six of the eleven participants did not complete all six online modules (see Table A), and only continued the in-person treatments. Opinions were divided on whether Back2Action might be the most optimal treatment to give psychologically-informed care: (1) Some found Back2Action highly beneficial and completed most digital health modules (P01-P03, P09, P10); (2) Others favoured discussing their psychological distress solely with their physiotherapist (P04 and P07); (3) Another participant (P06), expressed he did not feel that it was suitable to discuss his psychological distress with his physiotherapist. Nonetheless, this same participant and two others (P03 and P10) realized through Back2Action that they required specialized psychological treatment to resolve their psychological distress which contributed to their physical symptoms.

### 3.2 Theme 2: importance of the physiotherapist's active involvement in biopsychosocial blended care

In addition to participants' attitudes, the physiotherapist played an important role to encourage and support participants to complete the digital health modules and to deliver the intervention in a blended format. The manner in which the physiotherapist communicated the opportunity for participation in the RCT had an impact on the participants' motivation to initiate and maintain their engagement in Back2Action. Moreover, two participants (P06 and P07) who finished only one digital health module, mentioned that they lacked appropriate support of the physiotherapist, which diminished their motivation and established a sense of being unguided.

Others experienced positive support of the physiotherapist during the digital psychologically-informed digital health intervention. The in-person treatments and the written feedback motivated them to continue with the modules.

P01: “The feedback you then get afterwards [from the physiotherapist], because you're talking about it again, that feels like an encouragement [...], because if the physiotherapist says: ‘it's great that you have done all this’, then this motivates to continue with it”.

The physiotherapist also had a crucial role in implementing the obtained knowledge of the digital health modules into practice. The physiotherapist was able to personalise the information of the modules by building on specific topics and enabling participants to establish connections between their personal circumstances and the content presented in the digital health modules. Additionally, some physiotherapists ensured that the acquired knowledge was linked to exercises and/or experiences.

P03: “He [the physiotherapist] mainly repeated the information that was already discussed in the modules […] and then recommended exercises to show how I could relieve the pain myself.”

Despite the intended blended character of Back2Action (digital health and in-person physiotherapy), not all participants experienced the intervention as ‘blended’. In some cases, the content of the digital health was minimally or not discussed during the in-person treatments. Whereas some felt they lacked appropriate support of the physiotherapist, others did not consider this a hindrance; participant 2 completed six modules and perceived Back2Action as having two distinct components – physiotherapy and digital health, but did not express a need for additional guidance or involvement from the physiotherapist during the digital health modules.

P02: “My back and neck were completely stuck, so I went to the physiotherapist to loosen that, which helped. […] But I did not really talk to the physiotherapist about the content and assignments of the digital health modules […] I thought it [the modules] was more an eye opener for myself […] Through the modules I learned to deal more consciously with the stressors in my life.”

### 3.3 Theme 3: appreciation of digital health

Participants generally had a positive opinion about the design of the digital health modules. The combination of written information, videos and actively engaging in the assignments was experienced as meaningful. For example, participant 9 found recognition in the digital modules and felt accompanied by the stories of others. Also, the length of the modules and the reminders to start the next one were experienced positively.

P02: “… by answering questions from what you have just read, and then intentionally linking it back to yourself, made it really useful.”

However, there were also participants who indicated that the information in the modules was too tedious and lacked vivacity. For two participants (P07 and P04) these were reasons to stop after respectively one and four modules.

The concepts presented in the digital health modules that had the most profound impact on the participants were: ‘we have our own drug cabinet in the brain’, ‘pain and tissue damage rarely relate’, ‘pain depends on the balance of perceived danger and safety’ and ‘I can influence my pain by modifying my activities’. These insights influenced the way participants thought of their own body and improved their understanding about the origin and persistence of their health problem. Moreover, it also enhanced their ability to cope with their situation.

P04: “Especially in the beginning, it [the pain education] was a little bit confusing. […] But if you go a little deeper into it, you'll realise oh yes, that's precisely how pain, sensors, nerves and the brain work.”

A frequently mentioned helpful tool of the digital health modules was the weekly scheduling which was recurrent in multiple modules. The participants who used this tool to consciously plan meaningful activities, found this helpful to actually change their behaviour. A more structured daily life schedule provided them a better overview of their time with more intentional room for social or physical activities.

P09: “The weekly schedule forces you to do the activities […] you are therefore more likely to do them and it gives you an overview to see if you have completed your daily exercise or activities”.

### 3.4 Theme 4: trials and triumphs

Participants indicated that they achieved successes due to Back2Action. All participants mentioned that they made (sometimes small) changes in their daily routines during the treatment period, such as performing meaningful activities (e.g., reading a book, going for a walk, engaging in social activities). Some indicated they gained increased awareness in the connection between their physical and mental health due to the pain education which resulted in these changes in their daily routine. Others were motivated to be more active due to the behavioural activity assignments, such as the weekly scheduling, in the digital health modules.

P03: “By consistently doing activities, your mind gets distracted, which I found particularly interesting. And this is indeed the case, that when I play volleyball or take a walk, my pain is almost gone. Which I found especially interesting, by doing things I like, my pain becomes less.”

Participants stated that they could cope better with their pain. They perceived their pain as more bearable, were less guided by it and felt more in control of their symptoms. For example, one participant (P03) did not show a clinically relevant reduction in pain intensity, but experienced a good recovery. Others experienced enhanced awareness of themselves and their pain, leading to better self-understanding and improved coping skills.

P01: “The pain is not always less, but how I experience it is different […] Before (Back2Action) I used to react by doing less if I had pain, but now I am more aware, I can just manage it better, I am mentally stronger”.

Despite these gains, participants also voiced the difficulty to maintain these behavioural changes and implement these into long-term routines. Participants recognized that they consciously needed to make time to effectively work on and prioritise their recovery (e.g., take time to do their exercises, take rest, work on the digital health modules). However, stressful and busy periods in daily life, such as deadlines at work, moving house or the COVID-19 pandemic restrictions, or a flare-up of their pain could easily influence participants' recovery negatively. As a result, these obstacles made it challenging for participants to maintain a healthier lifestyle but also to continue the digital health modules.

P01: I began to feel a lot better, but now that my toe is broken, I suddenly find myself restricted. As a result, my mental state, back pain and ability to continue with Back2Action were affected.”

## 4 Discussion

This qualitative study was conducted to explore participants' experiences with the blended intervention “Back2Action” and to gain insights into the useful elements and areas that may require attention. The results showed that participants of Back2Action experienced positive changes: such as an increased awareness of the connection between physical and mental health (theme 1); along with small changes in daily routines and shifts in their thinking and pain coping mechanisms. Nonetheless, challenges persisted in altering long term routines (theme 4). Furthermore, the findings indicate that participants have diverse personal preferences for receiving blended biopsychosocial care, with not all participants experiencing the intervention as truly blended. Where theme 3 underscores the value of psychologically-informed digital health in enhancing better understanding of persistent pain, theme 2 emphasizes the significance of physiotherapist active involvement alongside this digital health (theme 2).

To our knowledge, Back2Action is the first intervention that combines psychologically-informed digital health care consisting of pain education and behavioural activation with regular in-person physiotherapy care. Back2Action has allowed individuals to better recognise signs of physical and mental strain (enhanced self-discovery), facilitating accurate interpretation of these signs and the ability to respect their limits without resorting to overly passive, avoidant or persistent coping strategies. These findings align with another qualitative study where patients with chronic pain experienced increased awareness of bodily signals and how pain was related to stress and activity, following a blended intervention consisting of psychomotor physiotherapy and digital health modules addressing informational and educational topics (Eiken et al., 2022).

A systematic review of digital health interventions showed that significant change in behaviour is observed following interventions which include education, goal setting and self-monitoring (Rose et al., 2017). These aspects were all incorporated in Back2Action. Additionally, the utilisation of various methods for information delivery, such as reading, video content, and interactive assignments within the digital health modules, alongside personalised in-person physiotherapy, likely facilitated to the perceived treatment effect of Back2Action. The sequential design of the modules facilitated repetition and deep learning which is crucial for behavioural change (Kwasnicka et al., 2016).

Drawing upon the experienced achievements stated by participants of Back2Action, we suggest that the integration of pain education could be enhanced by incorporating behavioural activation techniques. This aligns with a similar qualitative study on the experiences of individuals with chronic low back pain who received a multimodal treatment program, including elements of education and behavioural change techniques (Rizzo et al., 2023). This study also suggests that behavioural change techniques may facilitate self-management and treatment effectiveness (Rizzo et al., 2023).

Some participants did not show a clinically relevant difference in pain scores but experienced a good recovery, perceived their pain as more bearable and felt more in control of their symptoms. This highlights the complexity of defining a favourable outcome when assessing pain and reaffirms the value of qualitative research in exploring patient experiences within evidence-based practice.

Another main finding revolves around participants' diverse preferences in how the intervention should be delivered. Participants attitudes towards receiving psychologically-informed care varied, including choices such as with or without involvement of a physiotherapist, psychologist, or the use of digital health methods. Research both in physiotherapy and mental health suggests that digital or blended care may not be suitable for every patient (Wentzel et al., 2016; Koppenaal et al., 2022). Determining which individuals will benefit from digital or blended care, and what approach is most effective for each individual, is challenging (Du et al., 2020; Nicholl et al., 2017). A checklist could assist healthcare providers to assess the eligibility of blended care and ensure that the program aligns with the participant characteristics, their abilities, needs, and preferences (Kip et al., 2020).

Finally, our results underline the importance of the active role of the physiotherapist in encouraging and supporting participants with the digital part of Back2Action. This is achieved through reinforcing participant's competence via written feedback after each digital health module and through personalising the information during the in-person physiotherapy sessions. Research has shown that personalised, face-to-face support is crucial to achieve better outcomes and adherence in internet-based psychological interventions (Johansson and Andersson, 2012; Wilhelmsen et al., 2013). Also, a similar qualitative study on patient experiences with a blended biopsychosocial intervention found that blended interventions should be tailored to the individual situation and motivation status of the participants (Toonders et al., 2021).

In our study, some participants missed important personal support from the physiotherapist and the translation of the digital health modules to their personal needs, which might explain lack of adherence in completing all modules in some participants.

While dropouts and lack of adherence are common issues in online psychological interventions (Karyotaki et al., 2015), we expected better adherence in our blended intervention compared to solely digital health interventions. Possible explanations for this phenomenon include: (1) The 3-h training the physiotherapists received may have been insufficient for some physiotherapists to successfully guide a biopsychosocial blended intervention such as Back2Action, and (2) limited flexibility in digital health modules. Offering more flexibility in terms of module sequence, content and format could potentially offer greater benefits for individuals and increase adherence to digital health (Fernandes et al., 2022).

### 4.1 Methodological considerations

The credibility of our study is established through investigator triangulation, member checks, use of quotes and following the COREQ checklist (Tong et al., 2007). Through iterative data collection and analysis, data saturation and the uses of an emergent research design the dependability and credibility of our study is optimised. The confirmability is ensured using an audit trial, monthly peer debriefing and by discussing all findings in a constant, open, and reflective dialogue among all authors.

The transferability of our study is suboptimal, since we were compelled to use a convenience sample drawn from the experimental arm of an RCT, which evaluated the efficacy and cost-effectiveness of Back2Action. Participants' experiences with Back2Action and the RCT were inextricably linked. Participation in the RCT required the completion of additional questionnaires, which increased the burden on participants. This may have reflected negatively on participants' engagement and experiences with Back2Action. Although common practice (Wilhelmsen et al., 2013; Toonders et al., 2021; Eiken et al., 2022; Rizzo et al., 2023), based on our experiences, we recommend to conduct qualitative analyses separately from RCTs. Due to the inclusion of participants treated by different physiotherapists from various practices spread throughout The Netherlands and the variability in demographics characteristics, the transferability is supported.

The number of participants included in this study (*n* = 11) could be considered low; however, we believe saturation was reached as no new themes emerged after six interviews. Additionally, according to a systematic review, saturation in qualitative research can be achieved within a range of 9 to 17 interviews (Hennink and Kaiser, 2022). Although there was no purposive sampling for deviant cases, the entire spectrum of digital health completion was represented, including participants who completed all modules (*n* = 5) and those who completed only a few modules (*n* = 4). For these reasons, and by presenting the experiences of the participants in their context, we believe that the sample is suitable for ensuring the transferability of the data.

## 5 Conclusions

Participants of the blended biopsychosocial intervention experienced positive changes in thoughts and behaviours, including a better understanding of the connection between physical and mental health, better coping, and increased engagement in meaningful activities, which highlights the feasibility and acceptability of the blended intervention as a more holistic treatment within pain management. However, both continuing of the digital health and sustaining gains were perceived as challenging, particularly when facing life stressors. Additionally, it is important to highlight that not everyone experienced the intervention as blended.

This study contributes to our understanding of participants' experience in receiving biopsychosocial care and personal preferences in receiving musculoskeletal care. The various preferences for receiving psychologically-informed digital health poses challenges for implementation of biopsychosocial care in evidence-based practice.

## Ethics approval and consent to participate

Is mentioned in first paragraphs of methods.

## Funding

This research did not receive any specific grant from funding agencies in the public, commercial, or not-for-profit sectors.

## Declaration of competing interest

The authors declare that they have no known competing financial interests or personal relationships that could have appeared to influence the work reported in this paper.

## Appendix A Supplementary data

Supplementary material Image 1
